# Immunotherapy for neuroblastoma using syngeneic fibroblasts transfected with IL-2 and IL-12

**DOI:** 10.1038/sj.bjc.6603857

**Published:** 2007-06-26

**Authors:** S E Barker, S M Grosse, E K Siapati, A Kritz, C Kinnon, A J Thrasher, S L Hart

**Affiliations:** 1 Molecular Immunology Unit, UCL Institute of Child Health, 30 Guilford Street, London WC1N 1EH, UK

**Keywords:** neuroblastoma, fibroblasts, immunotherapy, cytokines, synthetic vectors

## Abstract

Cytokine-modified tumour cells have been used in clinical trials for immunotherapy of neuroblastoma, but primary tumour cells from surgical biopsies are difficult to culture. Autologous fibroblasts, however, are straightforward to manipulate in culture and easy to transfect using nonviral or viral vectors. Here we have compared the antitumour effect of fibroblasts and tumour cells transfected *ex vivo* to coexpress interleukin-2 (IL-2) and IL-12 in a syngeneic mouse model of neuroblastoma. Coinjection of cytokine-modified fibroblasts with Neuro-2A tumour cells abolished their *in vivo* tumorigenicity. Treatment of established tumours with three intratumoral doses of transfected fibroblasts showed a significant therapeutic effect with reduced growth or complete eradication of tumours in 90% of mice, associated with extensive leukocyte infiltration. Splenocytes recovered from vaccinated mice showed enhanced IL-2 production following Neuro-2A coculture, and increased cytotoxicity against Neuro-2A targets compared with controls. Furthermore, 100% of the tumour-free mice exhibited immune memory against tumour cells when rechallenged three months later. The potency of transfected fibroblasts was equivalent to that of tumour cells in all experiments. We conclude that syngeneic fibroblasts cotransfected with IL-2 and IL-12 mediate therapeutic effects against established disease, and are capable of generating immunological memory. Furthermore, as they are easier to recover and manipulate than autologous tumour cells, fibroblasts provide an attractive alternative immunotherapeutic strategy for the treatment of neuroblastoma.

Neuroblastoma is one of the most common solid tumours of childhood, accounting for 8–10% of all childhood cancers and 15% of cancer-related deaths (Young and Miller, 1975). The tumours may arise wherever neural crest cells occur, the most frequent locations being the adrenal medulla and sympathetic ganglia. Age of diagnosis is critical to outcome, and infants diagnosed in the first 12 months generally have a promising prognosis, even if suffering from metastatic disease. Those diagnosed over 1 year of age have a less favourable outlook and often fail to respond even to the most aggressive combinations of therapy. The current treatment for neuroblastoma patients with severe disease is usually chemotherapy, followed by surgical resection or radiotherapy (Pinkerton *et al*, 2000). Treatment for minimal residual disease involves high-dose chemotherapy with autologous stem cell rescue, or differentiation of the remaining malignant cells with retinoids (Philip *et al*, 1997). Patients with disseminated disease, however, frequently relapse, and so alternative treatments including immunotherapy are under investigation.

Immunotherapy using tumour cells transduced with single-chain interleukin-12 (IL-12) protein has shown promise in murine models of neuroblastoma (Lode *et al*, 1998b), an effect that was enhanced in combination with tumour-targeted interleukin-2 (IL-2) protein (Lode *et al*, 1999). In natural killer (NK) cells, IL-2 was shown to enhance the antitumour activity of IL-12 through the upregulation of IL-12 receptors and STAT4 (Wang *et al*, 2000). Interleukin-12 also induces CD25 expression on T cells, thereby enhancing their proliferation in response to IL-2 (Nguyen *et al*, 2000). We have demonstrated previously that Neuro-2A murine neuroblastoma cells could be transfected simultaneously with two plasmids using a synthetic vector system to express high levels of both IL-2 and single-chain IL-12 (Siapati *et al*, 2003). Transfection of Neuro-2A cells with IL-2 and IL-12 abolished their tumorigenicity in syngeneic A/J mice, and when injected into established tumours provoked an antitumour immune response involving CD8^+^ and CD4^+^ T cells, which led in some cases to complete tumour eradication.

Cytokine adjuvant immunotherapy strategies for neuroblastoma have been investigated clinically with both autologous and allogeneic neuroblastoma tumour cells transduced with IL-2 (Bowman L *et al*, 1998; Bowman LC *et al*, 1998). Autologous cells were much more effective in these studies, but the difficulty of establishing cultures of purified primary neuroblastoma tumour cells rapidly has limited the wider exploitation of this approach.

Fibroblast cultures from skin biopsies are relatively easy to establish and expand, and so autologous fibroblasts gene-modified to express cytokine adjuvants may offer a viable alternative to autologous tumour cells as cellular vaccines. Autologous fibroblasts transduced with IL-2 and mixed with autologous irradiated tumour cells have been used in phase I trials to treat colorectal carcinoma (Sobol *et al*, 1999), and IL-12-transduced autologous fibroblasts have been used to treat melanoma (Kang *et al*, 2001), both with promising results. However, so far there are no reports of autologous fibroblast therapy for neuroblastoma. In this study, we have compared the potential of IL-2- and IL-12-transfected murine syngeneic fibroblasts with IL-2- and IL-12-transfected tumour cells to generate an immune response in mice with an established tumour burden, and to establish protective immunity from tumour rechallenge.

## MATERIALS AND METHODS

### Cell culture

Murine neuroblastoma cells (Neuro-2A) (ATCC, Teddington, UK) were maintained in Dulbecco's modified Eagle's medium (DMEM)-Glutamax-1 (Invitrogen, Paisley, UK) containing 10% foetal calf serum (FCS) (Sigma, Poole, UK), 100 IU ml^−1^ penicillin, 100 *μ*g ml^−1^ streptomycin, 1% sodium pyruvate and 1% nonessential amino acids (Invitrogen, Paisley, UK). AJ3.1 is a mouse embryonic fibroblast line derived from A/J embryos 13.5 days postcoitum as follows: the crania and red organs were removed and the remaining tissue minced using scalpels, digested with trypsin, then passed through a cell strainer. The resulting suspension was plated in DMEM containing 10% FCS and antibiotics, with each embryo maintained as a separate culture. Cells were passaged every few days to maintain sparse colonies until the cultures reached senescence, and after 7–10 days growing colonies of cells were observed. One culture was selected for all further work and is hereafter referred to as AJ3.1.

### Plasmids

Human IL-2 gene was expressed from pCI-hIL-2 (Siapati *et al*, 2003). Murine IL-12 was expressed from the plasmid pCI-mIL-12, which was generated by subcloning into pCI (Promega, Southampton, UK) the gene encoding the single-chain fusion protein of the p35 and p40 subunits of murine IL-12 separated by a flexible linker, from plasmid pcDNA3.1scIL12 (Lode *et al*, 1998a). All plasmids were grown in *Escherichia coli* DH5*α* and purified using the EndoFree Mega plasmid kit (Qiagen, Crawley, UK).

### Transfections

All cells were transfected using the lipid : peptide : DNA plasmid (LPD) vector, as described previously (Hart *et al*, 1998). AJ3.1 cells were transfected using a peptide identified by phage display that binds to fibroblasts (gift from A Kritz, unpublished data). Neuro-2A cells were transfected with peptide 6 containing the *α*5*β*1 integrin-specific RRETAWA-binding domain isolated from a phage display library (Koivunen *et al*, 1994). The lipid used was Lipofectin (Invitrogen Ltd, Paisley, UK). All transfections were carried with a weight ratio of 0.75L : 4P : 1D in OptiMEM (Invitrogen, Paisley, UK) serum-free medium for 4 h before being replaced with complete medium.

AJ3.1 fibroblasts and Neuro-2A neuroblastoma cells were transfected with cytokine plasmids and supernatant was harvested for 5 days. Human IL-2 (hIL-2) and murine IL-12 (mIL-12p70) expression levels were determined by DuoSet ELISA Development System (R&D Systems, Abingdon, UK) according to the manufacturer's instructions.

For cells used in vaccinations, 3 × 10^6^ AJ3.1 fibroblasts were plated per 150 cm^2^ flask, and transfected for 4 h with LPD complexes containing 80 *μ*g DNA (40 *μ*g each of pCI-hIL-2 and pCI-mIL-12). 4 × 10^6^ Neuro-2A cells were plated per 150 cm^2^ flask and transfected with the LPD vector containing 80 *μ*g DNA. After 24 h, cells were harvested by trypsinisation and stored frozen until required for vaccination. On the day of use, cells were thawed, washed in RPMI medium before injection into the tumour.

### Animal vaccinations

All animal procedures were approved and licensed by the Home Office and performed to the standards required by the UK Co-ordinating Committee on Cancer Research (UKCCCR) (Workman *et al*, 1998). Female A/J mice of 5–7 weeks old were purchased from Harlan Laboratories (Oxford, UK). For tumorigenicity experiments, transfected or control AJ3.1 or Neuro-2A cells were trypsinised 24 h after transfection, washed and resuspended at 10^7^ cells ml^−1^. Mice were vaccinated by injection in the left posterior flank with 10^6^ wild-type Neuro-2A cells in combination with 10^6^ AJ3.1 cells, 10^6^ Neuro-2A cells transfected with IL-2 and IL-12 (Neuro-IL2/12) or 10^6^ AJ3.1 fibroblast cells transfected with IL-2 and IL-12 (AJ-IL2/IL12) (*n*=6 per group). Control animals received 10^6^ viable Neuro-2A tumour cells only. Tumour progression was monitored by measuring two perpendicular axes every 2–3 days using callipers. Mice were promptly culled if the tumour reached a diameter of 12 mm or was ulcerating. For vaccination experiments in mice with an existing tumour burden (*n*=16), mice were engrafted with 10^6^ unmodified Neuro-2A cells subcutaneously (s.c.) in the left posterior flank. On day 3 after injection of tumour cells, when tumours had reached approximately 2 × 2 mm, animals were vaccinated by intratumoral (i.t.) injection with 10^6^ Neuro-IL2/IL12, 10^6^ AJ-IL2/IL12 or by peritumoral injection if it was too difficult to inject inside the tumour. Control animals received 100 *μ*l RPMI (Invitrogen, Paisley, UK) medium only. All groups of mice received three doses of their vaccine, 2 days apart, on days 3, 5 and 7. Mice remaining tumour free 3 months after the initial injection were rechallenged with 10^6^ viable Neuro-2A cells in the opposite flank to the first injection and tumour progression was monitored. These rechallenged mice, which remained tumour free for a further 3 months, were culled and their splenocytes used for *in vitro* assays.

### Enzyme-linked immunosorbent spot assays (ELISpot)

Spleen cells were washed through cell strainers using RPMI to give single-cell suspensions. Cells were pelleted by centrifugation (240 × *g*) and erythrocytes were removed by lysis on ice for 5 min with 0.83% ammonium chloride. Remaining cells were then resuspended at 5 × 10^6^ cells ml^−1^ in RPMI, and then aliquoted into 96-well U-bottomed plates with 5 × 10^3^ Neuro-2A cells as targets at an effector : target (E : T) ratio of 100 : 1. Positive-control wells contained effector cells in 5 *μ*g ml^−1^ Concanavilin A and negative controls contained effector cells only. Plates were incubated overnight, then the contents of each well were transferred to 96-well MultiScreen Immobilon-P plates (Millipore, MA, USA) coated with 1 *μ*g ml^−1^ anti-mIL-2 (clone JES6-1A12; Pharmingen, San Diego, CA, USA), which had been blocked with FCS for 30 min and washed with wash buffer (PBS). Plates were incubated at 37°C and 5% CO_2_ for 24 h, then cells were washed off with wash buffer. Then, 2 *μ*g ml^−1^ biotinylated anti-mIL-2 (clone JES6-5H4, Pharmingen, San Diego, CA, USA) was added in wash buffer and incubated for 2 h, washed off and replaced with streptavidin-alkaline phosphatase (AP) conjugate (ExtrAvadin, Sigma, Dorset, UK) for 30 min. Spots were developed using AP substrate (BioRad, Hemel Hempstead, UK) and plates were read on a Bioreader 3000 LC plate reader (Biosys GmbH, Frankfurt, Germany).

### Lactate dehydrogenase release assay

Splenocytes prepared as above were plated into 24-well plates at 10^6^ cells ml^−1^ in RPMI containing 10% FCS, antibiotics and 60 IU rhIL-2 (Peprotech, London, UK) and cocultured with irradiated Neuro-2A cells at an E : T ratio of 50 : 1. After 4 days, a lactate dehydrogenase (LDH) assay was performed with CytoTox96 Non-Radioactive Cytotoxicity Assay kit (Promega, Southampton, UK). Cytotoxic T lymphocyte (CTL) effectors were cocultured in quadruplicate in RPMI-Glutamax-1 without phenol red (Invitrogen, Paisley, UK), containing 5% FCS and antibiotics in U-bottomed 96-well plates with 5 × 10^3^ viable Neuro-2A cells at an E : T ratio of 27 : 1 in 200 *μ*l. Spontaneous effector release controls were set up in quadruplicate. Plates were incubated for 5 h at 37°C, then 50 *μ*l supernatant was transferred to a flat-bottomed, 96-well plate and assay substrate added to each well. Plates were developed for 30 min and then read in a plate-reading Lucy 1 spectrophotometer (Anthos, Salzburg, Austria) at 492 nm and the percentage cytotoxicity was calculated using the formula: 
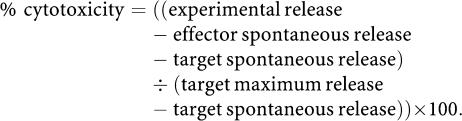


### Histological analysis

Tumours were excised from the same mice that were used for the LDH and ELISpot assay, fixed in 4% PFA, embedded in paraffin wax and 7 *μ*m sections mounted on polylysine-coated slides. For histological analysis, sections were serially rehydrated and stained with haemotoxylin and eosin, followed by serial dehydration with ethanol and mounting with glass coverslips. For immunostaining with anti-mouse-CD45 to determine the leukocyte infiltration, sections were serially dehydrated, then microwaved in antigen-unmasking solution for 10 min. Endogenous peroxidase activity was blocked (0.3% H_2_O_2_ (v v^−1^), 0.1% NaN_3_ (w v^−1^) in PBS) for 15 min, then slides were washed in PBS. Sections were blocked with PBS containing 10% rabbit serum (v v^−1^) (Vector Laboratories, Peterborough, UK) at room temperature (RT) for 30 min. Excess block was removed, then a 1 : 20 dilution of unconjugated rat anti-mouse CD45 (Pharmingen, San Diego, CA, USA) in PBS containing 1% BSA was applied. Sections were incubated overnight in a humid chamber at 4°C. Control slides were incubated with 10% rabbit serum in PBS. Sections were washed in PBS, then a 1 : 250 dilution rabbit anti-rat biotinylated antibody (Dako, Glostrup, Denmark) in PBS was added and incubated at RT for 30 min. Slides were washed and then incubated with a streptavidin-biotinylated peroxidase complex for 30 min (ABC Reagent, Vector Laboratories, CA, USA). Positively stained cells were identified with 3,3′-Diaminobenzidine (Sigma FAST DAB, Sigma Chemical Co., Dorset, UK). Sections were counterstained in Methyl Green (Vector Laboratories, CA, USA), washed twice in dH_2_O, dehydrated in butan-1-ol, then incubated in Histoclear for 10 min and air dried. Slides were mounted with glass coverslips and analysed with an AxioPhot 2 microscope (Zeiss, Welwyn Garden City, UK) attached to a ProgRes 3012 digital camera (Kontron Electronic, Chichester, UK) and images were analysed using Adobe Photoshop (Adobe Systems, San Jose, CA, USA).

### Statistical analysis

The two-sample Student *t*-test was used to assess the significance between experimental animal groups. Statistical significance was taken as *P*<0.05.

## RESULTS

### Abolished *in vivo* tumorigenicity of neuroblastoma cells when injected simultaneously with cytokine-transfected fibroblasts

First, the cytokine secreting potential of syngeneic fibroblasts was assessed. AJ3.1 fibroblast cells were transfected with pCI-hIL2 and pCI-mIL12 under optimised transfection conditions, and supernatants were harvested over 5 days, then secreted cytokines were quantified by ELISA. Peak cytokine levels from cytokine-transfected AJ3.1 cells (1100 ng 10^6^ cells^−1^ 24 h^−1^ of mIL-12 and 230 ng 10^6^ cells^−1^ 24 h^−1^ of hIL-2) were similar to those previously observed in transfected Neuro-2A cells (1350 ng 10^6^ cells^−1^ 24 h^−1^ mIL-12 and 150 ng 10^6^ cells^−1^ 24 h^−1^ hIL-2) (Siapati *et al*, 2003).

**Figure 1:**
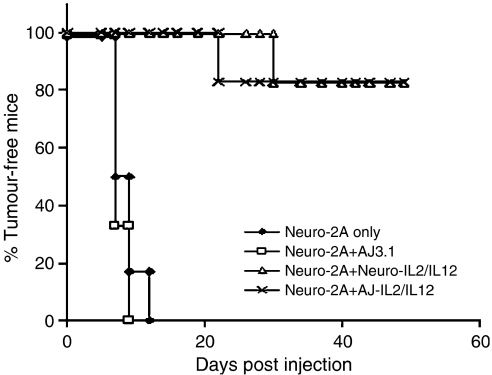
*In vivo* tumorigenicity of 10^6^ unmodified Neuro-2A cells when coinjected subcutaneously with 10^6^ unmodified AJ3.1, 10^6^ cytokine-transfected AJ3.1 cells or 10^6^ cytokine-transfected Neuro-2A in syngeneic mice (*n*=6 per group). In total, 83% of mice in the therapeutic groups did not develop tumours and remained tumour free for over 40 days, while control mice receiving unmodified AJ3.1+Neuro-2A or Neuro-2A only all developed aggressive tumours within 11 days.

For vaccine studies, a large population of cells was transfected, and stored frozen so that the same cellular vaccine was used in all experiments. A/J mice were injected s.c. in the left flank with 10^6^ untransfected Neuro-2A alone, or in combination with 10^6^ Neuro-IL2/IL12 cells, 10^6^ AJ-IL2/IL12 cells or 10^6^ untransfected AJ3.1 cells. All mice receiving untransfected Neuro-2A cells alone or in combination with untransfected AJ3.1 cells developed fast-growing tumours by days 11 and 9, respectively. Only one of six mice (17%) in each group treated with the Neuro-IL2/IL12 vaccine or the AJ-IL2/IL12 vaccine developed a tumour (Figure 1) and the other five in each group (83%) remained tumour free for over 40 days, a significant improvement over controls (*P*<0.01). Therefore, the antitumour efficacy of cytokine-transfected AJ3.1 fibroblast vaccine was equivalent to the Neuro-2A tumour cell vaccine.

### Effective treatment of established tumours with the AJ-IL2/IL12 vaccine

The most clinically relevant aspect of any tumour vaccine is the ability to mediate therapeutic responses against established tumours. Neuro-2A cells were engrafted in the left flank of A/J mice and when tumours were palpable (∼3 days), mice were vaccinated every 2 days for a total of three doses with Neuro-IL2/IL12 or AJ-IL2/IL12 cells (*n*=16 per group) or with RPMI medium (*n*=6), by i.t. injection. Each dose of cellular vaccine was from the same, single-transfected source. Six mice from each group having the biggest tumours were killed for *in vitro* analysis of the cellular immune response at 12 days post engraftment, which was the point when all the control mice had to be killed due to their tumour size. The other 10 mice from each therapeutic group were monitored for several months to study the long-term effect of vaccination on established tumours or killed if the tumour size reached a diameter of about 12 mm.

**Figure 2:**
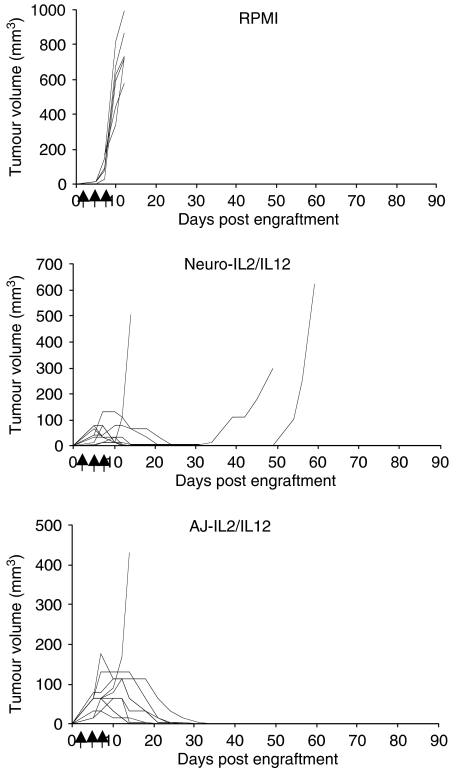
Effect of vaccination on established tumours. A/J mice were inoculated subcutaneously with 10^6^ wild-type Neuro-2A cells, and 3, 5 and 7 days later an equal number of Neuro-IL2/IL12 or AJ-IL2/IL12 cells or RPMI medium were injected intratumorally. Tumour growth of each animal (*n*=6 for the control group and *n*=10 for the therapeutic group) is represented by a single line.

In groups of mice (*n*=10) monitored for several months to study the long-term effect of vaccination on established tumours, only three of 10 mice (30%) in the group treated with the Neuro-IL2/IL12 vaccine and one of 10 mice (10%) in the group treated with the AJ-IL2/IL12 vaccine developed tumours (Figure 2). Seven mice (70%) of the Neuro-IL2/IL12 vaccine group and nine (90%) of the AJ-IL2/IL12 vaccine group remained tumour free for over 3 months, a significant improvement over the control group where 100% of the mice developed tumours and were killed at 12 days post engraftment (*P*<0.01).

**Figure 3:**
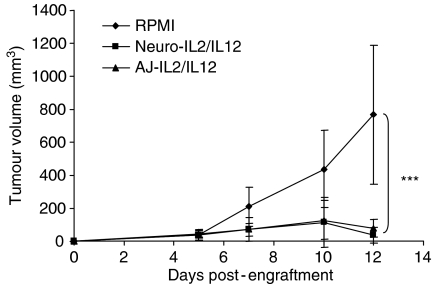
The average tumour volumes of mice bearing established tumours after treatment with three vaccines. A/J mice were engrafted with 10^6^ unmodified Neuro-2A cells and vaccinated with Neuro-IL2/IL12 or AJ-IL2/IL12 cells or RPMI medium on days 3, 5 and 7 directly into the tumour. By 12 days post engraftment, mice vaccinated with Neuro-IL2/IL12 or AJ-IL2/IL12 (*n*=16 per group) had significantly smaller tumours compared to RPMI-treated controls (*n*=6). Day 12 is the point when all the control mice have to be killed due to their tumour size. Significance was determined by *t*-test (^***^P<0.001).

Intratumoral treatment with the Neuro-IL2/IL12 vaccine resulted in significant tumour growth retardation compared to control mice receiving RPMI. Average tumour growth was reduced significantly by day 12 post engraftment to 36±47 mm^3^ compared to control mice receiving RPMI, where the average tumour volume was 767±423 mm^3^ (*P*<0.001) (Figure 3). In the group of animals where the AJ-IL2/IL12 vaccine was used to treat established tumours, a significant reduction in tumour growth was also observed at 12 days (81±55 mm^3^) compared with RPMI-treated controls (*P*<0.001) (Figure 3).

### Cellular immune response

**Figure 4:**
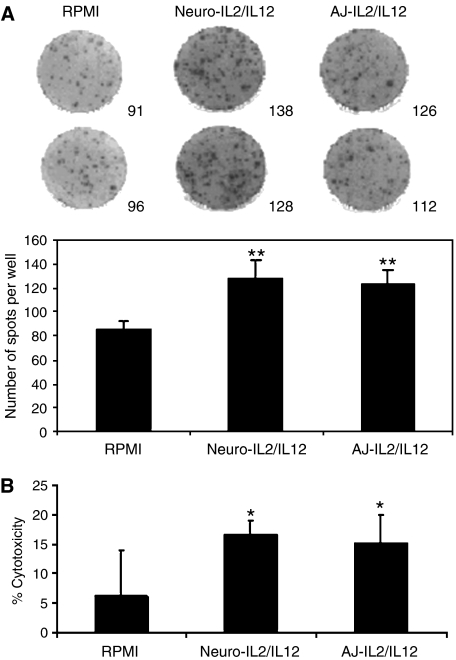
ELISpot and LDH release assays were performed using splenocytes from mice with treated established tumours. Mice were engrafted subcutaneously with tumour cells, then vaccinated 3, 5 and 7 days later. On day 12, six mice from each group were culled and splenocytes coincubated with Neuro-2A cells for 48 h hours. (**A**) Splenocytes from mice vaccinated with Neuro-IL2/IL12 or AJ- IL2/IL12 had significantly greater numbers of IL-2-expressing splenocytes than RPMI-vaccinated controls in response to Neuro-2A target cells (^**^P<0.01). Photographs are of representative wells and significance was determined by *t*-test. (**B**) LDH assay determined the cytotoxicity of splenocytes against Neuro-2A targets using a 27 : 1 E : T ratio. Mice vaccinated with Neuro-IL2/IL12 or AJ-IL2/IL12 showed significantly greater toxicity against Neuro-2A targets than splenocytes from mice vaccinated with RPMI. Significance was determined by *t*-test (^*^P<0.05).

The cellular antitumour immune response after vaccination was assessed by isolating splenocytes from six mice per group for *in vitro* analysis at 12 days post engraftment (Figure 4A). Splenocytes from mice receiving AJ-IL2/IL12 i.t. secreted high amounts of IL-2 (123±12 spots) in response to coculture with wild-type Neuro-2A cells. This result was similar to the number of spots produced by splenocytes from mice receiving Neuro-IL2/IL12 (128±15 spots). Both groups of vaccinated mice produced significantly more spots than RPMI-treated controls (*P*<0.01).

The cytotoxicity of CTL effectors was examined by LDH release from target Neuro-2A cells. Cytotoxic T lymphocytes, obtained by 4-day culture of splenocytes (*n*=6 per group) with irradiated Neuro-2A targets in the presence of rhIL-2, were cocultured with viable Neuro-2A for 5 h at an E : T ratio of 27 : 1 (Figure 4B). LDH released into the supernatant was assayed and used to calculate percentage cytotoxicity. Splenocytes from mice vaccinated i.t. with Neuro-IL2/IL12 showed significantly greater cytotoxicity (17±2.5%) compared to RPMI-treated controls (6±8%), *P*<0.05. Similarly, mice vaccinated i.t. with AJ-IL2/IL12 also exhibited greater cytotoxicity against Neuro-2A targets (15±5%) compared to RPMI-treated controls, *P*<0.05. Overall, there was no significant difference in cytotoxicity of CTLs from mice receiving either the Neuro-IL2/IL12 or AJ-IL2/IL12 vaccines.

These findings indicated that the antitumour efficacy of cytokine-transfected AJ3.1 fibroblast vaccine was as potent as the Neuro-2A tumour cell vaccine.

### Leukocyte infiltration and reduced vascularisation upon vaccination of established tumours

**Figure 5:**
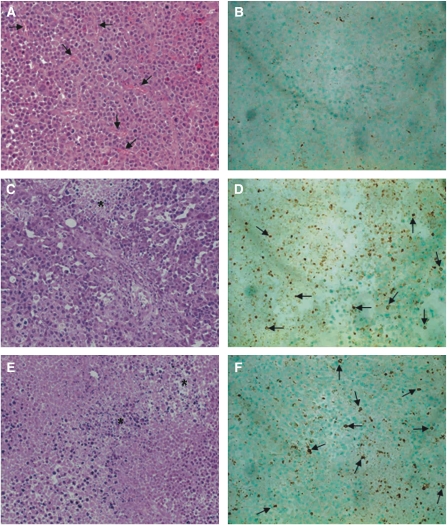
Tumour histology and immunohistochemistry. Mice vaccinated with RPMI showed well vascularised tumours (black arrow) (**A**) and negligible infiltration of CD45^+^ leukocytes (**B**). Tumours showed much reduced vascularity compared to RPMI-treated controls following vaccination with AJ-IL2/IL12 (**C**) or Neuro-IL2/IL12 (**E**) with areas of necrotic tissue (^*^). There was also extensive infiltration of CD45^+^ leukocytes (black arrow) in tumours treated with AJ-IL2/IL12 (**D**) or Neuro-IL2/IL12 (**F**). Representative sections are shown. Magnification × 40.

We then examined the histology of treated *vs* untreated tumours for differences in leukocyte infiltration or vascularisation. Sections from RPMI-treated control tumours were stained with haematoxylin and eosin, revealing highly vascularised regions with dense capillary networks (Figure 5A). Larger vessels were also present around the tumour periphery. Anti-CD45 staining showed negligible leukocyte infiltration of control tumours (Figure 5B). Interestingly, tumours from mice vaccinated intratumorally with AJ-IL2/IL12 (Figure 5C) or Neuro-IL2/IL12 (Figure 5E) suggested a decrease of capillaries within the tumour compared to RPMI-treated controls, with most vasculature confined to the tumour periphery. Necrotic areas could also be seen in the treated tumours, defined by punctate nuclear staining. Anti-CD45 staining demonstrated that tumours vaccinated intratumorally with AJ-IL2/IL12 (Figure 5D) or Neuro-IL2/IL12 (Figure 5F) had extensive infiltration of leukocytes, particularly around the tumour periphery. This contrasts with the absence of CD45^+^ cells in RPMI-treated control tumours (Figure 5B).

### Immunological memory against tumour cells of rechallenged mice

To establish if systemic immunological memory responses to tumour cells had been established, tumour-free mice (*n*=7 from Neuro-IL2/IL12 and *n*=9 from AJ-IL2/IL12 groups) were rechallenged 3 months after the initial vaccine with 10^6^ wild-type Neuro-2A cells in the opposite flank to the original injection, with no further vaccination. All mice in each group (100%) remained tumour free for at least 3 months.

ELISpot and LDH assays from these rechallenged mice showed similar results to those obtained with the vaccinated mice studied at 12 days post engraftment (Figure 4). All the splenocytes from these rechallenged mice released a high level of IL-2 after incubation with Neuro-2A cells and showed a strong cytolytic activity against Neuro-2A cells *in vitro*, with no significant difference between the vaccinated mice with AJ-IL2/IL12 or Neuro-IL2/IL12 cells (not shown). Therefore, the mice vaccinated with cytokine-transfected fibroblasts were protected as well as those vaccinated with Neuro-IL2/12 cells.

## DISCUSSION

Neuroblastoma immunotherapy using genetically modified tumour cells that secrete proinflammatory cytokines into the tumour environment is a promising approach for the treatment of the disease. However, difficulties in culturing sufficient quantities of primary tumour cells from surgical biopsies hamper these approaches. Autologous fibroblasts, genetically manipulated to secrete cytokines, offer an attractive alternative approach to cellular vaccines as they can be readily cultured and expanded from skin biopsies. They may be transfected with genes for proinflammatory cytokines and either injected directly into the tumour site or mixed with tumour cells for subcutaneous injection.

In this study, we have evaluated the potential of a vaccine consisting of syngeneic fibroblasts for immunotherapy in a murine model of neuroblastoma and compared this vaccine with a neuroblastoma tumour cell vaccine described previously (Siapati *et al*, 2003). The A/J mouse and syngeneic Neuro-2A cells are widely used by neuroblastoma researchers and they are considered to represent a good model of the disease (Ziegler *et al*., 1997). A metastatic model of diseases would be more clinically relevant, but at this stage of research, subcutaneous models are convenient and appropriate since tumours engrafted subcutaneously in the flank are accessible for measurement and treatment. The fibroblast vaccine consisted of an embryonic fibroblast cell line, AJ3.1, transfected with plasmids encoding IL-2 and IL-12, while the tumour cell vaccine comprised syngeneic Neuro-2A neuroblastoma cells transfected with the same plasmids. In previous studies, the tumorigenicity in A/J mice of Neuro-2A cells transfected with IL-2 and IL-12 (Neuro-IL2/IL12) was abolished, and Neuro-IL2/IL12 cells injected into established tumours generated an anti-neuroblastoma immune response, involving both CD8^+^ and CD4^+^ T cells, that led to tumour eradication in some mice (Siapati *et al*, 2003). The same cell-mediated mechanism of immunity with the same cytokines secreted by fibroblasts as syngeneic tumour cells is likely.

Expression of secreted IL-12 and IL-2 from transfected AJ3.1 cells peaked at 1100 ng 10^6^ cells^−1^ 24 h^−1^ and 230 ng 10^6^ cells^−1^ 24 h^−1^, respectively. This level of expression compared well with Neuro-2A cells where transfected levels of IL-12 and IL-2 were 1350 and 150 ng 10^6^ cells^−1^ 24 h^−1^, respectively. These levels had previously proved therapeutic for neuroblastoma in A/J mice (Siapati *et al*, 2003) and also compared well with levels of IL-2 and IL-12 expression reported with adenoviral vectors (Emtage *et al*, 1999). In that study, PyMT and Neu murine tumour cells derived from breast adenocarcinoma cells and transduced with Adenovirus-type 5 vectors produced 788 and 1204 ng 10^6^ cells^−1^ 24 h^−1^, respectively, of IL-12, and 32 and 51 ng 10^6^ cells^−1^ 24 h^−1^ of IL-2 (Emtage *et al*, 1999). These levels of adenovirus-mediated IL-2 and IL-12 expression were effective in mediating tumour regression in a murine model of breast cancer (Emtage *et al*, 1999). Therefore, IL-2 and IL-12 secretion levels from AJ3.1 cells transfected by the synthetic vector in this study were comparable to a viral vector system and were sufficient to anticipate an immunological antitumour effect. Indeed, the tumorigenicity of viable Neuro-2A tumour cells in A/J mice was significantly reduced when coadministered with AJ-IL2/IL12 and Neuro-IL2/IL12 vaccines, with five of six mice in each vaccine group failing to develop tumours.

Both the AJ3.1 and Neuro-2a vaccines significantly inhibited tumour growth when injected into small tumours compared with controls. Complete tumour growth inhibition was seen in seven of 10 (70%) mice treated with the Neuro-IL2/IL12 vaccine and nine of 10 (90%) mice treated with the AJ-IL2/IL12 vaccine. The tumour-free mice were rechallenged 3 months later by subcutaneous injection of viable Neuro-2A cells in the opposite flank to the original injection. All mice in each of these groups (100%) rejected this secondary challenge, suggesting that an immune memory response was induced by both types of vaccine.

The immune memory response in each vaccine group was assessed by IL-2 ELISpot of splenocytes following coculture with Neuro-2A cells, and CTL cytotoxicity against Neuro-2A target cells in LDH-release assays. Splenocytes from Neuro-IL2/IL12 and AJ-IL2/IL12 vaccinated groups generated similar number of spots, while control mice produced significantly fewer levels of spots. These findings suggested that the immune system of A/J mice recognised the tumour, and that the antitumour immune response in treated animals was sufficient to prevent tumour growth. After treatment with either the fibroblast or tumour cell vaccine, the cytotoxicity of CTLs in both vaccine groups was significantly higher than the CTL cytotoxicity from tumour-engrafted controls. This clearly indicated that cytokine adjuvant immunotherapy enhances CTL cytotoxicity, preventing tumour engraftment. Moreover, splenocyte activation and CTL cytotoxicity assays in these experiments suggested that fibroblasts are an efficient alternative to tumour cells for neuroblastoma vaccination.

Splenocytes from the RPMI control sample also appeared to recognise Neuro-2A cells, producing significant numbers of spots and were also able to lyse target cells. However, in each experiment, these controls were significantly less active than the vaccine groups indicating that the immune response raised was insufficient to limit tumour growth. This observation supports the strategy of immunotherapy for neuroblastoma, at least in this model, since although syngeneic, the Neuro-2A cells are recognised as foreign, perhaps by tumour-associated antigens.

Histological analysis of tumour sections showed extensive leukocyte infiltration in tumours from animals vaccinated with Neuro-IL2/IL12 or AJ-IL2/IL12 compared to negligible staining for leukocytes in control tumours, suggesting that the fibroblast vaccine recruits immune effector cells to the tumour site as effectively as the tumour cell vaccine. As well as mediating adaptive immune functions, IL-12 has angiostatic effects (Duda *et al*, 2000), and histological evaluation of tumours from mice treated with Neuro-IL2/IL12 or AJ-IL2/IL12 vaccines suggested that the vascularisation was reduced compared to control tumours. Necrosis of tumour tissue was another prevalent feature of tumours from vaccinated animals, possibly due to a combination of extensive infiltration of immune effector cells and the reduced vascular network, leading to a limited supply of nutrients and hypoxia.

Cytokine-expressing autologous tumour cells have generally only been used against the parental tumour, and similarly, allogeneic tumour cells have been used only to treat tumours of the same type. One potential major advantage of transfected fibroblasts over transfected tumour cells is that they may be effective against multiple tumour types. Autologous dermal primary fibroblasts transduced with a retrovirus to express IL-2 or IL-12 have already been used in clinical trials to treat two types of tumour (Sobol *et al*, 1999; Kang *et al*, 2001), and IL-2 engineered syngeneic fibroblasts have also been injected intracerebrally to treat experimental glioma (Lichtor *et al*, 2002).

Syngeneic fibroblasts have also shown promise as gene delivery ‘vehicles’ in a number of other experimental systems, such as delivery of IL-4 in collagen-induced rheumatoid arthritis (Bessis *et al*, 2002) and production of proteolipid protein to induce T-cell anergy in experimental autoimmune encephalomyelitis, the murine model of multiple sclerosis (Weiner *et al*, 2004). Primary fibroblasts have also been used to deliver BDNF and NT-3 in experimental models of spinal cord injury (Tobias *et al*, 2003).

In conclusion, cytokine-transfected autologous fibroblasts are a promising alternative to tumour cells for immunotherapy of neuroblastoma and other cancers.
